# An Umbrella Review of the Work and Health Impacts of Working in an Epidemic/Pandemic Environment

**DOI:** 10.3390/ijerph18136828

**Published:** 2021-06-25

**Authors:** Jonathan Fan, Sonja Senthanar, Robert A. Macpherson, Kimberly Sharpe, Cheryl E. Peters, Mieke Koehoorn, Christopher B. McLeod

**Affiliations:** 1 School of Population and Public Health, University of British Columbia, Vancouver, BC V6T 1Z3, Canada; robert.macpherson@ubc.ca (R.A.M.); kimberly.sharpe@ubc.ca (K.S.); mieke.koehoorn@ubc.ca (M.K.); chris.mcleod@ubc.ca (C.B.M.); 2 Cumming School of Medicine, University of Calgary, Calgary, AB T2N 1N4, Canada; cheryl.peters@ucalgary.ca; 3 Cancer Care Alberta, Alberta Health Services, Calgary, AB T2S 3C3, Canada; 4 CAREX Canada, Faculty of Health Sciences, Simon Fraser University, Vancouver, BC V6B 5K3, Canada; 5 Institute for Work & Health, Toronto, ON M5G 1S5, Canada

**Keywords:** systematic review, COVID-19, work and health, mental health and well-being, occupational health, pandemic

## Abstract

This umbrella review of reviews examined the evidence on the work and health impacts of working in an epidemic/pandemic environment, factors associated with these impacts, and risk mitigation or intervention strategies that address these factors. We examined review articles published in MEDLINE, PsycINFO and Embase between 2000 and 2020. Data extracted from the included reviews were analyzed using a narrative synthesis. The search yielded 1524 unique citations, of which 31 were included. Included studies were focused on health care workers and the risk of infection to COVID-19 or other respiratory illnesses, mental health outcomes, and health care workers’ willingness to respond during a public health event. Reviews identified a variety of individual, social, and organizational factors associated with these work and health outcomes as well as risk mitigation strategies that addressed study outcomes. Only a few reviews examined intervention strategies in the workplace such as physical distancing and quarantine, and none included long-term outcomes of exposure or work during an epidemic/pandemic. Findings suggest a number of critical research and evidence gaps, including the need for reviews on occupational groups potentially exposed to or impacted by the negative work and health effects of COVID-19 in addition to health care workers, the long-term consequences of transitioning to the post-COVID-19 economy on work and health, and research with an equity or social determinants of health lens.

## 1. Introduction

The effect of the coronavirus disease 2019 (COVID-19) pandemic on work, employment and health is considerable [1]. Among the working population, COVID-19 has posed a significant occupational health risk to workers in essential health care, service, manufacturing and agriculture industries [2,3,4,5]. In the United States, an estimated 10% of the workforce is potentially exposed to infection or disease more than once per week [2], with workers in health care and service jobs at greater risk of workplace exposure compared to high wage, knowledge-economy workers [2]. These work and health impacts are paralleled in recent epidemics and pandemics such as the H1N1 influenza pandemic of 2009–2010, the Ebola epidemic of 2013–2016, and the other coronavirus epidemics of Severe Acute Respiratory Syndrome (SARS) and Middle East Respiratory Syndrome (MERS) [6,7,8,9]. For example, during the H1N1 pandemic, health care providers globally faced a two-fold increase in the odds of influenza infection compared to non-health care provider control groups [9].

As the pandemic progresses globally, the direct and indirect economic and labour market consequences of COVID-19 have led to mass unemployment, increases in remote work and telework, and changes to other working arrangements and conditions in order to reduce exposure to COVID-19 for workers and the general public [10]. Low-wage, women and racialized workers have been disproportionally affected by the health, economic and work consequences of COVID-19 [4,10,11,12,13]. From February to April 2020, the employment rate in Canada fell by 38% among low-wage workers compared to 13% among all other paid employees [14].

Work during the COVID-19 pandemic has changed in various ways [1] and many of these changes are likely to persist, both in the mid-term and post-COVID-19 economy. Governments, public health agencies and occupational health regulators have responded to the economic and health risks posed by COVID-19 by providing enhanced income support for workers who have lost their jobs, expanding entitlement to sick leave, providing presumptive workers’ compensation coverage for COVID-19 infection for workers working in exposed environments, and creating policies and guidelines to reduce the risk of COVID-19 exposure in workplaces [15,16]. However, there is a need for actionable and targeted evidence that policymakers, employers, workers and other stakeholders can use to ensure that work is safe and healthy not only during the COVID-19 pandemic but also in its aftermath.

Recent editorials [1,4,10] have outlined research priorities that focus on keeping high-risk and essential service workers safe, as well as monitoring the consequences of the COVID-19 pandemic on the economic well-being of workers. Moreover, because the work and health effects of COVID-19 will have short, mid, and long-term consequences and will differ for younger versus older workers, it is imperative that a life course perspective be taken to understand the changing nature of work and health throughout the pandemic [17]. Although data from previous pandemics suggest that there are various social and occupational factors that modify health and well-being outcomes associated with working during a pandemic among health care workers [18], it is unknown if similar evidence exists for the broader working population.

While the medical and public health research community has transitioned to conducting COVID-19 research expeditiously, there are concerns about the quality of studies and duplication [19]. The volume of COVID-19 research being produced, much of it conducted without the usual checks and balances of the traditional research review process, presents a potential challenge to knowledge users and practitioners expecting to use this evidence to inform policy and practice. A key implication is that there is a need to strengthen the capacity around evidence appraisal and synthesis, including the ongoing monitoring of specific research areas [20]. An umbrella review of reviews typically yields the highest quality and most definitive body of evidence that can be used to provide input into the decision-making around research gaps and priorities [21].

We conducted an umbrella review of reviews published between 2000 and 2020 to summarize the evidence on the impacts of working during an epidemic or global pandemic on work and health outcomes (such as physical and psychological outcomes, risk of infection, disaster response and preparedness, and workers’ compensation outcomes such as return-to-work [RTW] following injury or illness), the socioeconomic, demographic and work factors that are associated with these outcomes, and possible risk mitigation or intervention strategies that address these factors or outcomes. The purpose of the summary is to inform evidence-based decision-making and best practices for the work and health of workers during an epidemic/pandemic. The purpose was also to identify research gaps to inform evidence needs for future studies and research funding priorities.

## 2. Materials and Methods

### 2.1. Design

Umbrella reviews build on the strengths of individual reviews by integrating the findings of multiple reviews together [21]. For the purposes of this umbrella review, working during an epidemic/pandemic was defined as any employment position that required a worker to work on or off-site from their usual place of work during an infectious disease epidemic/pandemic. This included working during the current COVID-19 pandemic as well as during other well-documented epidemics or outbreaks due to infectious diseases such as H1N1 influenza, MERS, SARS and Ebola. We examined the literature published during the 2000 to 2020 period to focus on recent pandemics/epidemics.

### 2.2. Systematic Search

We performed a search of MEDLINE, PsychINFO and Embase via the OVID interface for reviews published between 1 January 2000 and 13 July 2020. The search strategy was developed with the help of a trained occupational health librarian with expertise in conducting systematic reviews, reviewed by members of the research team for accuracy and piloted against a list of key publications that were known to the research team in advance. The search included all review types (e.g., systematic reviews, meta-analyses, rapid reviews, scoping reviews, realist reviews, narrative reviews), and used both key and MeSH terms related to work and health in an epidemic/pandemic. In addition to the search databases, the review was supplemented with hand searching of the medRxiv pre-print server for the health sciences [22], including all COVID-19 SARS-COV-2 pre-prints from medRxiv and bioRxiv identified with the search terms ‘COVID-19′ and ‘occupational health’. Full details of the search strategy, including MeSH terms, are in Appendix A.

### 2.3. Inclusion and Exclusion Criteria

Aspects of the Population, Intervention, Comparison, Outcome (PICO) framework [23] were used to define the research question and translate it into a set of study inclusion and exclusion criteria:

- Population: Adult working population (>15 years old) irrespective of occupation and nature of employment (e.g., full-time, part-time, temporary), not limited to any country or region;
- Intervention/Exposure/Phenomenon of Interest: Workplace exposure to respiratory pathogens or work within an epidemic/pandemic environment; factors associated with exposure and outcomes within an epidemic/pandemic environment; and possible risk mitigation or intervention strategies that address these factors. Studies that had an explicit focus on clinical best practices or surgical/clinical guidelines were excluded. Studies that focused on the specific effectiveness properties of certain types of PPE and infection control procedures were also excluded;
- Outcomes: We reviewed the work and health impacts of working during an epidemic or global pandemic, focusing broadly on physical and psychological outcomes; risk of infection; disaster response and preparedness; and workers’ compensation outcomes such as RTW following injury or illness. As a conservative measure, we did not limit the search strategy to specific types of work-health outcomes;
- Study design: We considered all review types of qualitative, quantitative or mixed methods research. To be eligible, the reviews had to conform to systematic and reproducible search protocols and contain a synthesis of findings.

Only articles published in English were included based on the language proficiency of the research team.

### 2.4. Screening

Screening of articles occurred in two phases. First, title and abstract screening were performed by two reviewers (J.F. and S.S.), with a random 10% sample checked by a third reviewer (C.M.). Disagreements on article relevancy were resolved by consensus with the research team. Eligible articles in the second phase, full-text review, were assessed by the same reviewers using a modified PICO framework for completeness before moving into data extraction.

### 2.5. Data Extraction

A data extraction tool was created by the research team based on elements from the Agency for Healthcare Research and Quality framework for identifying research gaps from systematic reviews [24], supplemented with elements from other review frameworks [17,25,26]. Two research team members (J.F. and S.S.) independently extracted information on the study objective, review type, number of studies included in the review, worker population, exposure/phenomenon of interest (determinants, barriers/facilitators, interventions), work and health outcomes, and main review findings as stated by the review authors. Extracted data from the reviews were discussed with the research team to ensure accuracy and relevancy to the research topic.

### 2.6. Critical Appraisal

Critical appraisal of included reviews was completed using the Joanna Briggs Institute (JBI) Checklist for Systematic Reviews [27]. The JBI checklist is not designed to assess the quality of individual primary studies but rather the methodological quality of the review using 11 items measured with either “yes”, “no” or “unclear”. These items include, for example, appropriateness of the search strategy and methods to combine studies, assessment of publication bias, and critical appraisal of included studies, with a summary score ranging from 0 to 11.

### 2.7. Data Synthesis

Synthesis involved comparing and contrasting the data extracted across the included reviews, to arrive at a consensus of the commonalities on the impact of an epidemic or pandemic on workers’ work and health outcomes, the factors that are associated with these work and health outcomes, as well as the intervention or risk mitigation strategies that address the exposure or phenomenon of interest, as concluded by the study authors. The research members further identified evidence gaps on the impacts of an epidemic/pandemic on work and health among workers in the literature that could inform future research.

## 3. Results

### 3.1. Search Strategy

**Figure 1:**
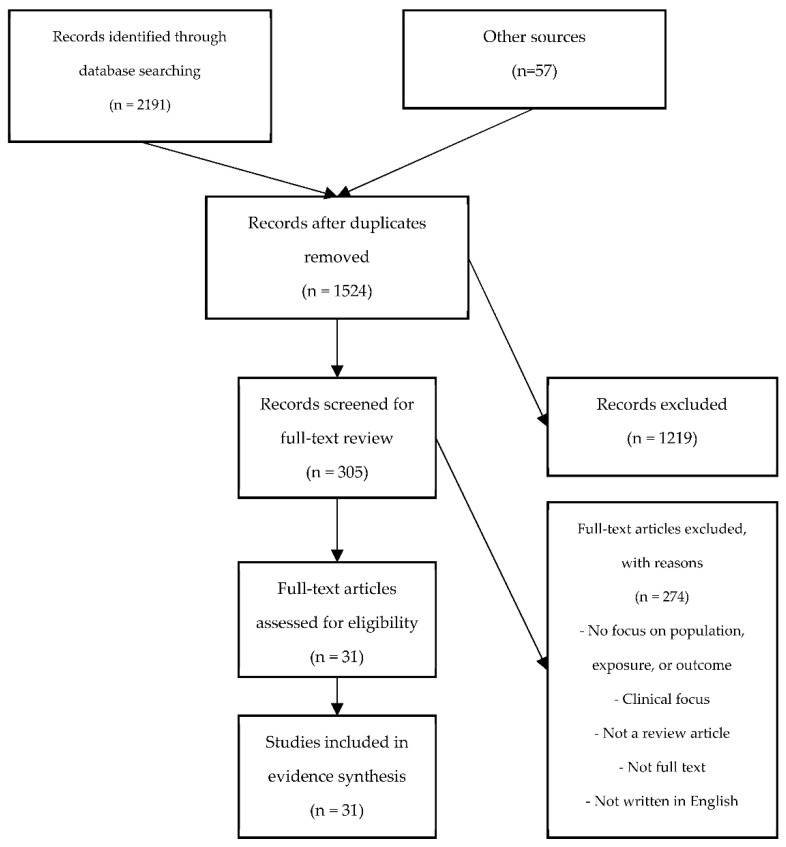
Flow diagram depicting the flow of information through the different phases of the scoping review.

A total of 2191 citations were retrieved from the databases. Deduplication using a combination of OVID and EndNote resulted in 1524 unique citations, of which 305 full-text articles were assessed for eligibility. In total, 31 reviews [6,7,8,9,18,28,29,30,31,32,33,34,35,36,37,38,39,40,41,42,43,44,45,46,47,48,49,50,51,52,53] met the final eligibility criteria for relevance to the work and health outcomes of working during an infectious disease epidemic/pandemic (Figure 1).

### 3.2. Review Characteristics

Table 1 presents an overview of characteristics for the included systematic reviews. The included reviews were published between 2005 and 2020, with a large proportion published in 2020 (*n* = 14, 45%). The average number of primary articles included in the reviews was 39, and the average number of items endorsed on the JBI Checklist for Systematic Reviews was 7.5 (out of 11), with a range of 4 to 10 items.

Almost all the reviews focused on health care worker (HCW) populations (*n* = 29), with only a few studies focusing on non-health care worker populations (*n* = 3) [39,40,51]. The health care worker reviews tended to focus on health care workers in general, although some included specific occupations, including physicians, paramedics, nurses and other health care workers.

Work and health outcomes included 15 reviews (48%) addressing mental illness and well-being and 13 reviews (42%) on infection risk. The remaining outcomes focused on willingness to respond or ability to work during an epidemic or pandemic (*n* = 9, 29%) and preparedness of systems or staff (*n* = 4, 13%). Reviews also examined multiple outcomes, including three reviews that examined mental health and well-being and risk of infection (*n* = 3, 10%); and one review that addressed mental health and well-being, risk of infection and willingness to work.

In terms of the main exposure or phenomenon of interest, there were 28 reviews (90%) focusing on exposure to infectious diseases in the health care work setting, 21 reviews (68%) focusing on the factors associated with exposure and work within an epidemic/pandemic environment, and 14 reviews (45%) focusing on intervention or risk mitigation strategies for study outcomes. Two-thirds of reviews included a mix of the aforementioned topics (*n* = 22, 71%).

### 3.3. Summary of Key Findings

The following section provides details on key findings across reviews related to the objectives. Gaps in research evidence are described, where identified.

#### 3.3.1. Work and Health Outcomes

Across the reviews, we found that a significant proportion focused on either mental illness and well-being outcomes, such as anxiety and posttraumatic stress [7,8,18,31,33,34,49,50,53], depression [6,7,31,50] and stigmatization [7,33,36], as well as outcomes related to the risk of infection to COVID-19 [6,7,32,43,49] or other respiratory illnesses. For example, a 2011 review by Koh et al. [36] examined health care worker risk perceptions towards emerging acute respiratory infectious diseases in health care settings. The authors found that health care workers perceived a high risk of personal and familial infection from SARS and stigmatization from the public. Similarly, a 2020 review by Bhaumik et al. [32] found that community health workers (CHWs) were at an increased risk of exposure to infectious disease due to a lack or incorrect usage of personal protective equipment (PPE), and that they faced stigmatization and isolation from communities they were serving as they were viewed as ‘carriers of infection’.

The studies that examined workers’ willingness to respond or ability to work in a public health event revealed a number of intersecting factors. For example, Connor [35] found that health care personnel considered four primary factors that either facilitated or hindered their intention to respond including the nature of the outbreak (e.g., how much is known about the illness), competing personal and professional obligations (e.g., concern for family versus duty to work), organizational role and climate, and knowledge and perceptions of efficacy as determined by years of practice or previous experience. Similar factors were found in the Lam et al. [48] study of nurses working during the SARS outbreak with the addition of the need for appropriate government policies and planning in place to address possible challenges and difficulties. Of the few reviews examining non-health care worker populations [39,51], the focus was on infection control. No reviews were found that examined RTW following workplace illness or workers’ compensation outcomes.

#### 3.3.2. Factors Associated with Work and Health Outcomes

The factors associated with work and health outcomes in an epidemic/pandemic environment can be broadly grouped into three categories encompassing individual, organizational and social factors.

Common individual factors across reviews included gender (being a woman) and having childcare obligations as influencing outcomes such as infection risk, psychological conditions or an unwillingness to respond or ability to work in an epidemic/pandemic event [6,8,30,34,36,37,38,44] while results were mixed for age. Particularly, some reviews concluded that younger age is suggestive of greater responsiveness to interventions to increase willingness to work during a pandemic [30] while others reported that younger HCWs were less willing to work [36] or were at increased risk of adverse psychological outcomes [34]. Neither race nor ethnicity was reported on, with the exception of one study [30].

Organizational factors encompassed the majority of reviews analyzed and ranged from issues with access to PPE and/or appropriate usage of PPE [6,18,32,34,37,38,42,45,49], a worker’s occupational role which was tied to the workplace risk environment (high vs. low risk environment) [6,8,18,30,34,35,36,37,42,44], a worker’s perception of safety and trust with the organizational climate [6,18,30,35,37,38,48], and organizational preparedness for an epidemic/pandemic whether through training or appropriate infection control practices (ICP) [6,8,18,30,32,34,35,36,37,38,45,48].

Across reviews, a worker’s occupational role, mostly within high-risk units (e.g., COVID-19 ward, emergency room) emerged as influencing health outcomes including risk of infection and mental health and well-being. Results were particularly consistent in indicating that physicians are less psychologically affected than nurses in facing an epidemic/pandemic. Yet, despite adverse outcomes, there was a strong ‘belief in duty’ across the studies where HCWs believed they had an obligation to care for a patient and this willingness indicated a greater risk acceptance in the course of an outbreak [30,48].

A worker’s perception of safety/trust and organizational preparedness typically referred to broader issues in the workplace, such as the culture and safety climate, to specific policies and procedures, such as policies around preparedness for an outbreak [37,38]. Among the reviews, the importance of information concerning disease outbreak situations and ICP were highlighted, regardless of the type and scale of epidemic/pandemic. For example, a review by Lam et al. [48] on the SARS and H1N1 outbreaks found that working during novel outbreaks induced confusion and uncertainty and forced nurses, for example, to use their clinical judgement in making decisions for patients. Similarly, reviews repeatedly mentioned the need for clear communication from management and training and education to workers yet there was little information regarding which formative training and education strategies are effective for managing outbreaks and adhering to ICP.

Finally, regarding social factors, reviews revealed concerns about the challenge of weighing work with safety for family and friends [8,30,34,35,36,44,45] but also societal stigma because of working with infected patients [18,32,34,36,45,49]. Common coping mechanisms to withstand stigmatization included support from family and peers [18,34,48] and seeing their efforts translate to patients getting better [34].

#### 3.3.3. Risk Mitigation or Intervention Strategies

The role of interventions was discussed in a selection of reviews [6,18,30,32,34,36,37,39,42,45,47,49,50], with a focus on addressing mental health outcomes and infection risk during a pandemic. For example, the review by Brooks et al. [18] proposed a list of recommendations for protecting the mental health of HCWs during outbreaks of infectious diseases, including providing appropriate specialized training; encouraging supervisor and co-worker support; ensuring adequate communication; ensuring mental health support measures are in place; developing occupational health policies or support systems; developing educational interventions and coping mechanisms to manage fear and stigma of infection; developing interventions to emphasize the potential positive effects of working in a crisis; and providing web-based support or discussion groups to reduce social isolation. However, although specific guidelines around these interventions had been proposed in the reviews, there was no emergence of a consensus and limited evidence of effectiveness. For example, the review by Gómez-Durán et al. [50] concluded that HCWs experienced considerable psychological distress, substance abuse, and stigma during quarantine. The authors also noted that suitable alternative accommodation and personalized monitoring during quarantine may be useful intervention measures to prevent adverse effects in health care workers. However, the authors pointed to the need to develop a consensus on core psychological interventions when HCWs undergo quarantine. Finally, while general population reviews have demonstrated the effectiveness of non-pharmaceutical interventions, such as physical distancing and quarantine [16,54], only a few of the included reviews in our study examined their implementation within worker populations, albeit as a secondary focus.

#### 3.3.4. Reviews Examining a Combination of the Aforementioned Topics

These reviews [6,18,30,32,34,36,37,42,47,49] primarily reported on the mental health and well-being of workers and/or infection risk as work and health outcomes; individual, organizational and social factors related to these outcomes and only secondarily examined risk mitigation strategies. The majority of reviews offered high-level risk mitigation and intervention strategies, such as increasing personal compliance with infection prevention and control and providing flexible work hours to reduce risk of infection or providing appropriate services (psychological intervention programs) to reduce distress in HCWs. A few exceptions went one step further to translate the combined findings into a set of recommendations for protecting the health and well-being of workers [18,34,36,49].

## 4. Discussion

The objectives of this umbrella review were to summarize the evidence on the impacts of working during an epidemic/pandemic environment on work and health outcomes, the factors that are associated with these outcomes, and risk mitigation or intervention strategies that address these factors or outcomes. The umbrella review also aimed to identify research gaps to inform evidence needs for future studies and research funding priorities. Based on a review of 31 reviews, we found that there was a significant focus on health care workers as a worker population. The reviews identified a variety of individual, social and organizational factors that influenced work and health outcomes associated with working during a pandemic/epidemic environment among health care worker populations. Only a few reviews focused on other worker populations or on the implementation of intervention strategies in the workplace.

Current research to date can be characterized by an extensive focus on HCWs, their risk of infection, attempts to improve PPE or its use in health care settings, and a focus on ameliorating the negative mental health consequences for HCWs working in an epidemic/pandemic exposed environment. Not surprisingly, HCWs face a greater risk of infection when working in pandemic-exposed environments and report a variety of common short-term and long-term adverse mental health outcomes including anxiety, posttraumatic stress and depression. However, there are other occupations that face an increased risk of exposure on a frequent basis, including essential workers in service, manufacturing and agriculture jobs [2,3] as well as labourers across sectors (e.g., construction, oil and gas, processing/manufacturing) [3]. The available published primary research that examines non-health care workers tends to examine infection risk, COVID-19 incidence, COVID-19 related mortality or psychological effects in either the general worker populations or in specific worker populations such as meat-packing or processing plants [55] as well as essential workers including social care and transport workers, although these specific populations were not identified in any systematic reviews as of the date of the last search.

Given the unique health and economic context of the pandemic, including potential labour shortages coupled with the need to ensure adequate protection for workers [56,57,58], future research should examine outcomes beyond initial infection risk, as well as the key barriers, enablers and mitigation strategies that drive differences in these outcomes [59]. Future research should also examine these factors in relation to other occupation and industry groups that are underrepresented in the literature. The research evidence on COVID-19 is evolving rapidly and we anticipate that additional reviews on COVID-19 research will continue to be published [60]. To a limited extent, the research gap on non-health care workers is slowly being filled with current or planned research, including primary research studies, and this area of research is expected to grow with the priority research competitions hosted by national and international health agencies [20].

Evidence yielded by the reviews on the important questions of factors related to work and health outcomes were multifaceted. Being female and having childcare obligations were negatively associated with willingness to report to work during an epidemic/pandemic. These are likely interrelated in that women are typically responsible for childcare but also represent a majority of the health care workforce, especially nursing staff, who may have a disproportionate risk of exposure and infection in their role [44]. Taken together, women’s primary responsibility for childcare may limit their ability to work both within and outside the home during an epidemic/pandemic but also for reasons of concern for the safety of children from exposure to infection brought in from the parental workplace.

The use of PPE (N95 or surgical masks, gloves, gowns) and careful donning and doffing of PPE were consistently identified as mitigating the risk of infection to workers and important to HCWs willingness to report to work. While the efficacy of certain types of PPE and disinfection procedures were not the focus of this umbrella review, a substantial number of worker populations suggested that major PPE shortages interfered with a healthy working environment. This was a challenge identified in the Chersich et al. (2020) review on HCWs in Africa [49], for example, which found that shortages contributed to greater stress and anxiety, suggesting the need for a more equitable distribution of PPE especially in low to middle-income countries. Meanwhile, lack of compliance with PPE use was primarily tied to inadequate training around PPE, especially in smaller scale hospitals with limited infrastructure, large surges of patients, and not enough staff to provide care [45]. Further, while training and education around PPE and other ICP were mentioned, they were poorly defined across studies. One reason for this may be that the nature of outbreaks is variable and evolving. As well, the majority of included primary studies in the reviews used cross-sectional surveys which may not be appropriate in investigating training and education. Thus, there appeared to be a general trend where responsibility was placed on the worker to stay safe and there was a gap in research calling for informed guidelines and best practices for future pandemics.

Only a few studies of HCWs compared and contrasted occupational roles to find that nurses are more likely to experience adverse health outcomes compared to physicians. Possible explanations for these results are varied. First, this could potentially be due to the fact that nurses are repeatedly engaged and in close proximity with patients compared with physicians [18]. Additionally, physicians could be protected from adverse health outcomes as a result of longer and more specific training (e.g., resilience training) [8]. To alleviate workloads/demands and burden of care, some of the proposed initiatives include mobilizing community and volunteer HCWs, drawing on retired HCWs, or fast-tracking medical students, although these are rarely implemented likely due to cost [61,62]. Meanwhile, social stigma and rejection associated with working in a high-risk environment seemed to affect workers irrespective of their occupational role. The World Health Organization (WHO) has produced some guidance for media to minimize stigmatization (e.g., highlighting the effectiveness of prevention measures rather than focusing on individual behaviours) [63], but a firmer approach may be needed to protect the psychological well-being of workers.

Finally, reviews that offered the most relevant or actionable information to policymakers focused on effectiveness of interventions to improve or support HCWs mental health, as well as reviews that identified specific factors that influenced health care infection risk, use of PPE and HCWs’ willingness to respond or ability to work in pandemic or natural disaster environments. Some evidence suggests that workplace interventions need to be targeted towards modifiable risk factors at the individual and organizational level [59,64]. For example, recent studies have found that job security [56] and perceived adequacy of PPE and workplace-based infection control procedures [57] may be important variables that influence the risk of worse mental health symptoms. Future research should continue to explore how these factors and others identified in the included reviews can be targeted to address mental health and well-being concerns as workplaces re-open or continue to re-open [59], as well as evaluation studies that examine their effectiveness over time.

### 4.1. Recommendations for Future Research

This umbrella review found that, while there is research evidence related to the impacts of working on work and health outcomes during an epidemic/pandemic, there are also considerable gaps that should be considered in future work to provide a more complete picture.

First, there is a need for research that considers the long-term consequences of transitioning to the post-COVID-19 economy on work-related health outcomes. The current umbrella review found no reviews with studies on RTW or work disability outcomes within the context of a pandemic, for example. Creating safe and healthy workplaces in a post-COVID-19 economy lies at the intersection of public health, occupational health, workers’ compensation and labour standards [10,65]. For example, in Canada, public and occupational health officials recommend or require that individuals, including workers, who are experiencing COVID-19 like symptoms stay home and avoid exposure to others [15,16,65]. However, access to sick leave may be governed by provincial standards and most workers may not have access to employer-paid sick leave [65]. Therefore, associated risks and the ability to foster safe and healthy workplaces could vary by workplace and by work setting. Workplaces may also face new challenges in accommodating disabled or injured workers during a global pandemic and during the economic recovery phase with fewer restrictions. Moreover, the ability to work from home may vary by income level, occupation group or industry setting [65]. This creates the potential for novel collaborative opportunities in which employers, occupational health agencies and private insurers could work together to accommodate workers with disabilities in the workplace while complying with broader human rights legislation.

Second, equity considerations were only tangentially referenced in the current research assessed in this umbrella review despite the fact that the economic and public health response to COVID-19 has exposed and amplified work-related inequities related to job loss and reduced work hours for precarious, low wage workers, women and racialized workers [10,11,12,13]. Furthermore, those who are able to continue to work are more likely on the front line as essential workers with a greater risk of exposure to COVID-19 [2,13]. These categories of workers may face challenging decisions related to continuing to work for financial security reasons while facing elevated risks to worker health while doing so. Moving forward, equity considerations should be adopted as a principle of research funding and evidence use.

Finally, due to the predominance of research around specific outcomes including mental health and well-being and risk of infection from working in a pandemic-exposed environment, we recommend that future work focus on the effectiveness of intervention and/or risk mitigation approaches, which may be able to address questions about effective guidelines to improve workplace safety that spans working during an epidemic/pandemic to the resumption of on-site work in the case of fewer economic restrictions [10]. Moreover, real-world evidence on the effectiveness of potential risk mitigation or intervention strategies, within the context of the current pandemic, could aid in the preparation for future pandemics beyond COVID-19 [66,67,68].

### 4.2. Strengths and Limitations

To the best of our knowledge, this is the first umbrella review to examine the evidence on the impacts of working during an epidemic/pandemic on work and health outcomes, the factors that are associated with these outcomes, and potential risk mitigation or intervention strategies that address these factors or outcomes. This umbrella review synthesized a vast amount of information in a short time with a robust methodological approach that included: (1) an inclusive and wide-ranging search, (2) no restrictions on worker population, review design and research themes around work and health, and (3) critical appraisal measures to provide the best available evidence on work and health impacts during an epidemic/pandemic.

However, this umbrella review does need to be considered in light of a few limitations. First, due to a large number of citations, especially the abundance of COVID-19 related research, we did not assess more general research that may have applicability to worker populations, such as the effectiveness of masks or other face coverings in reducing the transmission of COVID-19 [69], exposure and contamination routes (e.g., aerosol-generating procedures, open versus laparoscopic procedure), as well as the efficacy of vaccination in working populations. Second, in the appraisal of the reviews against the JBI checklist, we found that the “publication bias” item scored the lowest on the majority of included reviews that may skew the evidence base (e.g., over-representing health outcomes). As well, primary studies might be included in more than one review and this may further over-emphasize evidence in a particular occupation, such as health care. We did not include grey literature although database searches were combined with searches in the medRxiv and pre-print server for the health sciences to ensure a complete record of articles. Lastly, our paper does not account for reviews of 2020 COVID-19 primary research that will likely be published in the coming months. It may be important to consider these papers to identify research gaps and inform evidence needs for future studies.

## 5. Conclusions

Our umbrella review found a large volume of reviews on mental health and well-being and infection risk to health care workers; common factors that contribute or protect against adverse health; and a limited evidence base on effective mitigation strategies. Combined together, this umbrella review is able to provide timely input into the decision-making around research gaps and priorities [20]. Particularly, the review identified the need for research on occupational groups that are potentially exposed to or impacted by the negative work and health effects of COVID-19 in addition to health care workers, research on the long-term consequences of transitioning to the post-COVID-19 economy on work and health, and research with an equity or social determinants of health lens. As the COVID-19 pandemic continues in 2021 and beyond, we anticipate that the number of relevant work and health studies and systematic reviews will increase substantially in volume and hope that these recommendations are considered to provide a broad picture of work and health impacts of working during a global pandemic.

## Figures and Tables

**Table 1:** (**A**) Characteristics of included systematic reviews that examined infection risk outcomes. (**B**) Characteristics of included systematic reviews that examined mental illness and well-being outcomes. (**C**) Characteristics of included systematic reviews that examined disaster response and preparedness outcomes. (**D**) Characteristics of included systematic reviews that examined a mix of outcomes.

(**A**)						
**#**	**Ref.**	***n***	**Objectives**	**Population**	**Exposure/Phenomenon of Interest**	**Outcome Category**
1	Ahmed et al. 2018	15	Review of studies examining the role of social distancing in non-health care workplaces in reducing influenza transmission.	Non-health care workplaces	General work exposures	Infection risk
					Interventions/risk mitigation (social distancing)	
2	Hofmann et al. 2020	72	Review of studies examining the burden of NoV outbreaks on staff and implications for future prevention strategies.	General employees	General work exposures	Infection risk
3	Lietz et al. 2016	26	Review of studies examining the occupational risk of influenza A (H1N1) infection among health care personnel during the 2009 pandemic.	HCW	Working in health care setting	Infection risk
4	Mhango et al. 2020	11	Review of studies examining COVID-19 infection risk factors among HCW.	HCW	Working in health care setting	Infection risk
					Barriers and enablers of study outcomes	
5	Moore et al. 2005	168	Critical review of literature examining the organizational and individual factors that protect HCWs from infectious diseases at work.	HCW	Working in health care setting	Infection risk
					Barriers and enablers of study outcomes	
					Interventions/risk mitigation	
6	Selvaraj et al. 2018	94	Review of studies examining infection and mortality rates and common exposure risks among HCW during Ebola and Marburg virus outbreaks.	HCW	Working in health care setting	Infection risk
7	Thomas et al. 2017	8	Review of studies examining the impact of infectious disease on health outcomes among paramedics.	HCW	Working in health care setting	Infection risk
					Barriers and enablers of study outcomes	
					Interventions/risk mitigation	
8	Yassi et al. 2005	Not stated	Review of studies examining the organizational, environmental, and individual factors that influence the success of infection control and occupational health programs in relation to SARS and other respiratory pathogens; and important factors as identified by HCWs.	HCW	Working in health care setting	Infection risk
					Barriers and enablers of study outcomes	
(**B**)						
**#**	**Ref.**	***n***	**Objectives**	**Population**	**Exposure/Phenomenon of Interest**	**Outcome Category**
9	Brooks et al. 2018	22	Review of studies examining social and occupational factors affecting the psychological wellbeing of HCWs involved in the SARS epidemic.	HCW	Working in health care setting	Mental illness and well-being
					Barriers and enablers of study outcomes	
					Interventions/risk mitigation	
10	Gardner et al. 2015	20	Critical review of the psychological impact of SARS among survivors.	HCW	Working in health care setting	Mental illness and well-being
11	Gómez-Durán et al. 2020	12	Review of studies examining the psychological impact of quarantine on HCWs.	HCW	Interventions/risk mitigation (working in quarantine setting)	Mental illness and well-being
12	Kisely et al. 2020	59	Review of studies examining the psychological effects on clinicians of working to manage novel viral outbreaks and successful measures to manage stress and psychological distress.	HCW	Working in health care setting	Mental illness and well-being
					Barriers and enablers of study outcomes	
					Interventions/risk mitigation	
13	Luo et al. 2020	62	Review of studies examining the psychological and mental impact of the COVID-19 pandemic among HCWs, the general public and patients with pre-existing conditions or COVID-19.	HCW	Working in health care setting	Mental illness and well-being
					Barriers and enablers of study outcomes	
14	Pan et al. 2020	7	Review of studies examining the anxiety status of Chinese medical workers during the COVID-19 pandemic.	HCW	Working in health care setting	Mental illness and well-being
15	Pappa et al. 2020	13	Review of studies examining the prevalence of depression, anxiety and insomnia among HCWs during the COVID-19 pandemic.	HCW	Working in health care setting	Mental illness and well-being
					Barriers and enablers of study outcomes	
16	Preti et al. 2020	44	Review of studies examining the psychological impact of epidemic/pandemic outbreaks (i.e., SARS, MERS, COVID-19, Ebola, influenza A) on HCWs.	HCW	Working in health care setting	Mental illness and well-being
					Barriers and enablers of study outcomes	
17	Spoorthy et al. 2020	6	Review of studies examining the literature on mental health problems faced by HCWs during the COVID-19 pandemic.	HCW	Working in health care setting	Mental illness and well-being
					Barriers and enablers of study outcomes	
18	Vyas et al. 2016	32	Review of studies examining the potential psychological impact of deploying in support of the U.S. response to Ebola in West Africa.	HCW/military	Working in health care setting	Mental illness and well-being
					Barriers and enablers of study outcomes	
(**C**)						
**#**	**Ref.**	***n***	**Objectives**	**Population**	**Exposure/Phenomenon of Interest**	**Outcome Category**
19	Aoyagi et al. 2015	41	Estimate the proportion of HCWs willing to work during an influenza pandemic and identify associated risk factors.	HCW	Working in health care setting	Willingness or ability to work
					Barriers and enablers of study outcomes	
20	Connor et al. 2014	70	Review of studies examining the factors associated with the intention of healthcare personnel to respond to uncommon events, such as a natural disaster or pandemic.	HCW	Working in health care setting	Willingness or ability to work
					Barriers and enablers of study outcomes	
21	Devnani et al. 2012	32	Review of evidence examining the willingness of healthcare personnel to work during an influenza public health emergency.	HCW	Working in health care setting	Willingness or ability to work
					Barriers and enablers of study outcomes	
					Interventions/risk mitigation	
22	Rossow et al. 2014	28	Review of evidence examining HCWs’ willingness to report to work during an influenza pandemic.	HCW	Working in health care setting	Willingness or ability to work
23	Kunin et al. 2013	10	Review of studies examining the challenges faced by general practitioners when participating in pandemics or epidemics across countries.	HCW	Working in health care setting	Health system preparedness
					Barriers and enablers of study outcomes	Willingness or ability to work
24	Lam et al. 2018	7	Review of studies examining the core components that constitute nurses’ preparedness in an epidemic event.	HCW	Working in health care setting	Health system preparedness
					Barriers and enablers of study outcomes	Willingness or ability to work
25	Pincha Baduge et al. 2018	20	Review of studies examining emergency department and emergency nurses’ preparedness for management of Ebola outbreaks.	HCW	Working in health care setting	Health system preparedness
					Barriers and enablers of study outcomes	Willingness or ability to work
					Interventions/risk mitigation	
26	Puig-Asensio et al. 2020	35	Review of studies examining the benefits and challenges of Ebola epidemic preparation among hospitals in developed countries during the 2014–2016 Ebola epidemic.	HCW	Working in health care setting	Health system preparedness
					Barriers and enablers of study outcomes	Willingness or ability to work
					Interventions/risk mitigation	
(**D**)						
**#**	**Ref.**	***n***	**Objectives**	**Population**	**Exposure/Phenomenon of Interest**	**Outcome Category**
27	Bhaumik et al. 2020	36	Rapid evidence synthesis on roles, barriers and enablers for COVID-19 prevention and control among community health workers.	HCW	Working in health care setting	Infection risk
					Barriers and enablers of study outcomes	Mental illness and well-being
					Interventions/risk mitigation	Willingness or ability to work
28	Chersich et al. 2020	32	Review of studies examining the infection risks and mental health challenges that HCWs face in the COVID-19 pandemic and propose interventions to counter these in Africa.	HCW	Working in health care setting	Infection risk
					Barriers and enablers of study outcomes	Mental illness and well-being
					Interventions/risk mitigation	
29	Chou et al. 2020	64	Review of studies examining the burden of SARS-CoV-2, SARS-CoV-1, and MERS on HCWs and risk factors for infection, using rapid and living review methods.	HCW	Working in health care setting	Infection risk
					Barriers and enablers of study outcomes	Mental illness and well-being
					Interventions/risk mitigation (PPE and infection control)	
30	de Pablo et al. 2020	115	Review evidence of the impact of SARS/MERS/COVID-19 on physical and mental health outcomes among HCWs.	HCW	Working in health care setting	Infection risk
						Mental illness and well-being
31	Koh et al. 2011	14	Review of studies examining HCWs’ perceptions of risk from exposure to emerging acute respiratory infectious diseases and the perceived effectiveness of strategies used to facilitate healthy coping in acute hospital and community healthcare settings.	HCW	Working in health care setting	Infection risk
					Barriers and enablers of study outcomes	Mental illness and well-being
					Interventions/risk mitigation	

HCW = Health care worker.

## Data Availability

Not applicable.

## Appendix A

**Table A1:** Search strategy performed on the MEDLINE, PsychINFO and Embase databases via OVID.

	**Work Terms**
1	work/
2	(work or worker? or worksite? or workplace? or work-related$ or ((work or working or job) adj (related$ or environment? or site? or place? or status$))).ti,ab.
3	workplace/
4	job-related.ti,ab.
5	occupation$ related.ti,ab.
6	exp Health personnel/
7	(“healthcare worker *” or “health care worker *” or “healthcare provider *” or “health care provider *” or “hospital employee *” or “hospital staff” or “hospital personnel” or nurse$ or doctor$ or physician$ or “general practitioner *” or “healthcare support worker *”).ti,ab.
8	exp employment/
9	(employee? or employer? or employment).ti,ab.
10	staff.ti,ab.
11	(telework * or telecommut * or “remote e-work *” or “home-based work *” or “home-based telework *” or “working from home” or “remote work *” or “remote employee *” or “remote office” or “e-work *” or “distance work *” or “mobile work *”).ti,ab.
12	(labo?r or labo?rer).ti,ab.
13	(occupational or occupation?).ti,ab.
14	occupations/ or exp occupational diseases/ or occupational health/ or occupational exposure/ or occupational injuries/ or accidents, occupational/ or industrial accidents/
15	((occupat * or job or work *) adj2 (safety or exposure * or injur * or accident * or disease *)).ti,ab.
16	occupational hygiene.ti,ab.
17	Sick Leave/
18	absenteeism/
19	Presenteeism/
20	presenteeism.ti,ab.
21	productivity.ti,ab.
22	(work$ adj2 injur$).ti,ab.
23	(work$ adj2 (disable? or disabilit$)).ti,ab.
24	(return$ adj3 work$).ti,ab.
25	(stay$ adj3 work$).ti,ab.
26	exp workers’ compensation/
27	Work * compensation.ti,ab.
28	exp “rehabilitation, vocational”/
29	(Unemployment or underemployment).ti,ab.
30	(“job loss *” or “loss of work”).ti,ab.
31	((burnout or “burn out” or strain or stress or “mental health”) adj2 (employ * or occupat * or job or work * or vocation * or profession *)).ti,ab.
	**Disease Terms**
32	exp Coronavirus/ or exp coronavirus infections/
33	(coronavirus * or coronovirus * or coronavirinae * or CoV or ((corona * or corono *) adj1 (virus * or viral * or virinae *))).ti,ab,kw,kf.
34	disease outbreaks/ or epidemics/ or pandemics/
35	((disease$ adj2 outbreak$) or epidemic$ or pandemic$).ti,ab.
36	Severe Acute Respiratory Syndrome/
37	(“severe acute respiratory syndrome *” or SARS).ti,ab,kw,kf.
38	(“SARS-CoV-1” or “SARSCoV-1” or “SARSCoV1” or “SARS-CoV1” or SARSCoV or SARS-CoV or SARS1 or “SARS-1” or SARScoronavirus1 or “SARS-coronavirus-1” or “SARScoronavirus 1” or “SARS coronavirus1” or SARScoronovirus1 or “SARS-coronovirus-1” or “SARScoronovirus 1” or “SARS coronovirus1”).ti,ab,kw,kf.
39	2003 SARS.ti,ab.
40	Middle East Respiratory Syndrome Coronavirus/
41	(“middle east respiratory syndrome *” or “middle eastern respiratory syndrome *” or MERSCoV or “MERS-CoV” or MERS).ti,ab,kw,kf.
42	exp Ebolavirus/
43	Ebola *.af.
44	(H1n1 or (swine flu and pandemic?) or (flu and pandemic?) or (influenza and pandemic?)).ti,ab.
45	Flavivirus Infections/ or Flavivirus/
46	zika.mp.
47	Zika Virus Infection/ or Zika Virus/
	**Work and Disease Terms**
48	1 or 2 or 3 or 4 or 5 or 6 or 7 or 8 or 9 or 10 or 11 or 12 or 13 or 14 or 15 or 16 or 17 or 18 or 19 or 20 or 21 or 22 or 23 or 24 or 25 or 26 or 27 or 28 or 29 or 30 or 31
49	32 or 33 or 34 or 35 or 36 or 37 or 38 or 39 or 40 or 41 or 42 or 43 or 44 or 45 or 46 or 47
50	48 and 49
	**Limit to Systematic Reviews**
51	50 use ppezv
52	limit 51 to systematic reviews
53	limit 50 to systematic review
54	Systematic Review/ or “Systematic Reviews”/
55	(“systematic review” or “scoping review” or “rapid review” or “integrative review” or “meta-analysis” or “realist review” or “thematic review” or “narrative review” or “narrative synthesis” or “best evidence synthesis” or “umbrella review” or “critical review” or “literature review” or “review of review?”).ti,ab.
56	50 and (54 or 55)
57	52 or 53 or 56
	**Other Limits**
58	limit 57 to english language
59	limit 58 to yr = “2000 -Current”
60	remove duplicates from 59
61	59 use ppezv
62	exp animals/ not humans/
63	61 not 62
64	59 use emczd
65	(exp animal/ or exp nonhuman/) not exp human/
66	64 not 65
67	59 not (61 or 64)
68	63 or 66 or 67
69	remove duplicates from 68
